# Chronic treatment of (R)‐*α*‐lipoic acid reduces blood glucose and lipid levels in high‐fat diet and low‐dose streptozotocin‐induced metabolic syndrome and type 2 diabetes in Sprague‐Dawley rats

**DOI:** 10.1002/prp2.306

**Published:** 2017-04-03

**Authors:** Hardik Ghelani, Valentina Razmovski‐Naumovski, Srinivas Nammi

**Affiliations:** ^1^ School of Science and HealthWestern Sydney UniversityNew South Wales2751Australia; ^2^ National Institute of Complementary Medicine (NICM)Western Sydney UniversityNew South Wales2751Australia; ^3^ South Western Sydney Clinical SchoolSchool of MedicineUniversity of New South WalesNew South Wales2052Australia

**Keywords:** (R)‐ *α* ‐lipoic acid, high‐fat diet, hyperglycemia, hyperlipidemia, metabolic syndrome, streptozotocin, type 2 diabetes

## Abstract

(R)‐ *α* ‐lipoic acid (*ALA*), an essential cofactor in mitochondrial respiration and a potential antioxidant, possesses a wide array of metabolic benefits including anti‐obesity, glucose lowering, insulin‐sensitizing, and lipid‐lowering effects. In this study, the curative effects of *ALA* (100 mg/kg) on a spectrum of conditions related to metabolic syndrome and type 2 diabetes (*T2D*) were investigated in a high‐fat diet (HFD)‐fed and low‐dose streptozotocin (STZ)‐induced rat model of metabolic syndrome and *T2D*. The marked rise in the levels of glucose, triglycerides, total‐cholesterol, LDL‐cholesterol, and VLDL‐cholesterol in the blood of HFD‐fed and low‐dose STZ‐injected rats were significantly reduced by *ALA* treatment. Furthermore, *ALA* treatment significantly increased the serum HDL‐cholesterol levels and tended to inhibit diabetes‐induced weight reduction. Mathematical computational analysis revealed that *ALA* also significantly improved insulin sensitivity and reduced the risk of atherosclerotic lesions and coronary atherogenesis. This study provides scientific evidence to substantiate the use of *ALA* to mitigate the glucose and lipid abnormality in metabolic syndrome and *T2D*.

**Abbreviations**
ACOX: acyl‐CoA oxidase
AI: atherogenic index
ALA*α*: (R)‐ *α* ‐lipoic acid
AMPK: adenosine monophosphate activated protein kinase
ATGL: adipose triacylglycerol lipase
CRI: coronary risk index
DHLA: dihydrolipoic acid
FAS: fatty acid synthase
GDH: glucose dehydrogenase
GLUTs: glucose transporters
GPO: glycerol‐phosphate oxidase
HDL: high‐density lipoprotein
HFD: high‐fat diet
LCAT: lecithin cholesterol acyl transferase
PPAR: peroxisome proliferator‐activated receptor
STZ: streptozotocin

## Introduction

Metabolic syndrome is described as a multi‐faceted disorder incorporating obesity, impaired fasting glucose and/or impaired glucose tolerance, reduced insulin sensitivity and dyslipidaemia that predisposes type 2 diabetes (*T2D*) (Alberti et al. 2006). As a major high‐risk factor of *T2D*, metabolic syndrome poses a significant health burden around the world (Grundy 2012). According to World Health Organisation, 422 million people are affected with *T2D* by 2014 and this number is expected to rise to 592 million by 2035 (WHO, 2016). The modern lifestyle incorporating a high‐calorie diet and decreased energy expenditure also contributes to the pandemic of metabolic syndrome and *T2D* (Aude et al. 2004). Epidemiological studies also revealed that 90% of all patients with *T2D* are or have been overweight, and indicated that obesity is a strong risk factor and cause of *T2D* and associated metabolic disturbances (Kahn et al. 2006). It is also estimated that up to 58% of *T2D* cases can be prevented in the high risk (pre‐diabetes) population if proper preventive measures are taken (Ada 2004).

The events of hyperglycemia and hyperlipidemia and their association present a constellation of high‐risk factors in the development of diabetic and cardiovascular complications (Lender and Sysko 2006). To reduce these serious complications, a multi‐targeted approach controlling blood glucose and lipids is needed (Moller 2001). The currently available therapeutic options such as exercise, dietary modification, or a combination of synthetic anti‐diabetic, and hypolipidaemic drugs possess their own limitations and a multitude of undesirable side effects (Lender and Sysko 2006). Therefore, single medicinal agents having dual properties are in great demand. This has led to continued efforts to explore the effectiveness of new therapeutic substances from natural sources for the control of metabolic syndrome and *T2D*. In particular, supplementation of health‐promoting nutraceuticals such as naturally occurring bioactive compounds that are capable of eliciting therapeutic responses at the targeted cellular level may be an effective and convenient strategy to improve overall health and reduce disease risk. In addition, nutritherapy could prove to be a powerful alternative to pharmacotherapy due to its relative safety, cost‐effectiveness, and ability to modulate specific and sometimes multiple molecular targets (Davi et al. 2010).

Alpha‐lipoic acid (*ALA*; 1, 2‐dithiolane‐3‐pentanoic acid), also known as thioctic acid, is traditionally recognized as an essential cofactor in mitochondrial respiratory enzymes that catalyze the oxidative decarboxylation reactions in the body (Packer et al. 1995). Chemically, *ALA* is a short‐chain fatty acid with a disulphide group in its dithiolane ring and a chiral carbon resulting in *R* and *S* enantiomers. Although the majority of the commercially produced *ALA* consists of a racemic admixture, the R form is the biologically active form that is endogenously produced by the body while the S form is produced from chemical manufacture and is not biologically active (Shay et al. 2009). At the cellular level, *ALA* is reduced to dihydrolipoic acid (*DHLA*), which has a number of cellular actions including free radical scavenging and modulating oxidative stress and inflammatory pathways (Shay et al. 2009). *ALA* when exogenously administered is readily absorbed from the gut and has been clinically used in Europe for the treatment of diabetic polyneuropathy (Biewenga et al. 1997). *ALA* has been shown to possess anti‐oxidant, cardiovascular, cognitive, anti‐ageing, detoxifying, anti‐inflammatory, anti‐cancer, and neuroprotective pharmacological properties (Goraca et al. 2011).

In laboratory experiments, the effect of *ALA* on glucose and lipid levels has been investigated both in vitro and in vivo. Daily administration of *ALA* has been shown to protect the development of diabetes in cyclophosphamide‐induced diabetic mice (Faust et al. 1994). In addition, chronic supplementation of *ALA* has been shown to lower blood glucose in normal (Nagamatsu et al. 1995), streptozotocin‐ (Khamaisi et al. 1999; Salama 2011; Arambasic et al. 2013; Dinic et al. 2013), and alloxan‐induced diabetic‐ (Anuradha and Varalakshmi 1999; Sudheesh et al. 2011), and in fructose‐fed rats (Castro et al. 2013) with conflicting results on blood insulin levels and insulin sensitivity. Conversely, others reported that *ALA* elicited no effect on blood glucose and glucose tolerance in normal (Khamaisi et al. 1995), fructose‐fed (Black et al. 1998), streptozotocin‐induced diabetic rats (Nagamatsu et al. 1995; Black et al. 1998; Maritim et al. 2003; Wang et al. 2013; Jin et al. 2014) as well as in high‐fat diet (HFD)‐fed rabbits (Chen et al. 2009) and in genetically induced diabetic rats (Feng et al. 2013; Midaoui et al. 2015).

The lipid‐lowering response of *ALA* has also been studied in various animal models. The supplementation of *ALA* has been shown to lower weight gain and plasma total and LDL cholesterol and/or triglyceride levels in normal (Huong and Ide 2008) and in genetically induced diabetic rats (Sena et al. 2008; Butler et al. 2009; Carrier et al. 2014) as well as in HFD‐fed rats (Sena et al. 2008; Yang et al. 2008; Timmers et al. 2010; Seo et al. 2012; Miao et al. 2013), mice (Yi and Maeda 2006; Jang et al. 2014), rabbits (Chen et al. 2009), and in streptozotocin‐induced diabetic rats (Budin et al. 2007). In addition, *ALA* has been shown to protect arterial lesion formation in vitro (Lee et al. 2012). Conversely, no changes in serum total cholesterol and/or triglyceride levels were observed in streptozotocin‐induced diabetic rats (Salama 2011) and in HFD‐fed obese Zucker rats (Carrier et al. 2014; Rideout et al. 2016). *ALA* has also been shown to increase high‐density lipoprotein (HDL) cholesterol levels in high‐fat diet‐fed rats (Miao et al. 2013).

One of the rationales for this study is stemmed from the observed discrepancies in the previously reported in vitro and in vivo studies of *ALA*. Indeed, a majority of these studies either did not provide information on which type of *ALA* (whether a racemic admixture or a particular *ALA* enantiomer) was used or varied in a wide sub‐therapeutic doses of *ALA*. Secondly, although a large body of the above reported work on *ALA* has been carried out in numerous available animal models (spontaneous/chemical‐/dietary‐induced), the pattern of disease establishment and progress in most of them is not similar to the clinical situation of metabolic syndrome and *T2D* in humans. The recommended human dose of *ALA* in metabolic syndrome is approximately 14 mg/kg which is equivalent to a rat dose of approximately 100 mg/kg based on body surface area (McNeilly et al. 2011; Nair and Jacob 2016). Therefore, in this study, we investigated the in vivo effects of R‐*ALA* (a bioactive enantiomer) at a dose of 100 mg/kg for its glucose and lipid regulating activities in HFD‐fed and low‐dose streptozotocin (STZ)‐treated rat model which replicates the natural history and metabolic characteristics of human metabolic syndrome and *T2D*.

## Materials and Methods

### Chemicals used

(R)‐ *α* ‐Lipoic acid and streptozotocin were purchased from Sigma (St. Louis, MO, USA). The *Accuchek* ^®^ *Active* blood glucose meter with glucose test strips and *Accutrend Plus* ^®^ Instrument (for blood cholesterol and triglycerides) with cholesterol and triglycerides test strips were purchased from Roche Diagnostics, Mannheim, Germany. The HDL cholesterol assay kit was purchased from Wako Diagnostics, Japan. All other chemicals used were of analytical grade.

### Animals and diets

Twenty‐nine (29) adult male Sprague‐Dawley (SD) rats (350–450 g) obtained from the Animal Resources Centre (Canning Vale, WA, Australia) were used in the studies. Upon arrival, the rats were randomly housed in polypropylene cages (3 per cage) to minimize isolation stress. The animal facility was well‐ventilated and maintained at an ambient temperature of 24 ± 2°C, relative humidity of 50–60% with 12‐h light and dark cycle. The rats were acclimatized to the laboratory conditions for 1 week prior to experimentation and provided with standard diet and water ad libitum. Both the standard (AIN93G) and high‐fat (SF02‐006) rat pellet diets were supplied by Speciality Feeds (Glen Forrest, WA, Australia). The standard diet contained (in weight percentage) approximately: 69% carbohydrate, 19.6% protein, 4.6% fat, 4.8% crude fiber; and the high‐fat diet contained 10.6% carbohydrate, 19.4% protein, 60% fat, and 4.7% crude fiber. The use and care of the animals in this experimental protocol was approved by the Institutional Animal Care and Ethics Committee (Approval Numbers: A10296 and A11259) of the Western Sydney University following the NHMRC guidelines on Australian code of practice for the care and use of animals for scientific purposes.

### Experimental design and treatments

**Figure 1:**
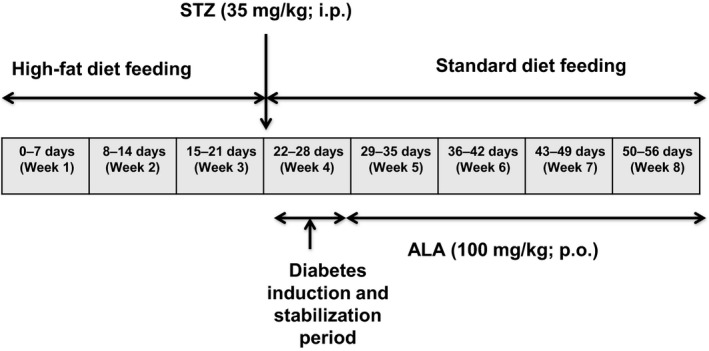
Schematic representation of experimental protocol. ALA, Alpha lipoic acid; STZ, Streptozotocin; i.p, intraperitoneal; p.o., per oral.

The rats were weight‐matched and divided into three groups (non‐diabetic control, diabetic control and *ALA*‐treated). The non‐diabetic control rats (*n* = 5) were fed with standard diet throughout the experiment, while the diabetic control (*n* = 12) and *ALA*‐treated rats (*n* = 12) were fed with HFD for 3 weeks. After 3 weeks of dietary intervention, the diabetic control and the *ALA*‐treated rats were fasted overnight and injected with a single intraperitoneal low‐dose of freshly prepared STZ (35 mg/kg dissolved in 0.1 mol/L citrate buffer; pH 4.5), while the non‐diabetic control rats received 1 mL/kg of 0.1 mol/L citrate buffer. The diabetic control and the *ALA*‐treated rats were then switched to the standard diet for the rest of the study and allowed to drink 5% glucose solution to counteract hypoglycemia due to sudden outbursts of insulin from the pancreatic *β* cells during the first 24 h after the STZ injection. All the animals were observed after the STZ or placebo injections. After 1 week, when the diabetic condition was stabilized, the STZ‐treated diabetic control and *ALA*‐treated rats with random blood glucose above 250 mg/dL were considered as diabetic and used in the study. The rats in the *ALA*‐treated (*n* = 9) group were administered with 100 mg/kg of *ALA* by oral gavage once daily for 4 weeks, while the diabetic control (*n* = 10) and non‐diabetic control (*n* = 5) groups received the vehicle (1% sodium CMC) by oral gavage once daily for 4 weeks as schematically depicted in Figure 1.

### Blood collection and experimental parameters

After 4 weeks of *ALA* treatment, the rats were fasted overnight for 12 h and blood sampling was done from the lateral saphenous vein under a conscious condition in all groups of rats. The blood samples were either immediately subjected to biochemical analysis or were allowed to clot for 30 minutes before being centrifuged at 850*g* for 15 min. The serum was separated and stored at −20°C until biochemical analysis.

### Body weight data

The weekly body weights were recorded in all the groups of rats between 9 am and 10 am and continued throughout the study.

#### Determination of serum glucose

Serum glucose was measured with an *Accuchek* ^®^ Active blood glucose meter and test strips using an enzymatic reflectance photometric assay based on the glucose dehydrogenase (GDH) method following manufacturer's instructions. Briefly, one drop of fresh venous blood was applied to the reagent area of a test strip and, when prompted, the strip was inserted into the glucose meter test chamber that directs light onto the test area. The glucose in the sample reacts with the reagents in the strip pad causing a color change. The amount of light reflected from the colored test area is proportional to the concentration of glucose measured by the photometer and is converted into a digital readout.

#### Determination of serum triglycerides and total cholesterol

Serum triglycerides and total cholesterol were measured using an *Accutrend Plus* ^®^ Instrument and test strips using an enzymatic reflectance photometric assay based on the glycerol‐phosphate oxidase (GPO) and cholesterol oxidase methods respectively following manufacturer's instructions. Briefly, one drop of fresh venous blood was applied to the reagent area of a test strip and, when prompted, inserted into the test chamber of the instrument that directs light onto the test area. The triglycerides/total cholesterol in the sample reacts with the reagents in the strip pad causing a color change. The amount of light reflected from the colored test area is proportional to the concentration of triglycerides/total cholesterol measured by the photometer and is converted into a digital readout.

#### Determination of TyG‐index

Triglyceride‐glucose (*TyG*)*‐*index levels, as a measure of insulin sensitivity (Guerrero‐Romero et al. 2010) was calculated from the real‐time fasting serum glucose and triglyceride concentrations of different groups of rats using the mathematical *TyG* formula:

TyG‐index=ln[(fasting triglycerides (mg/dL)×fasting glucose (mg/dL)/2)]

#### Determination of serum HDL‐cholesterol

Serum HDL‐cholesterol was estimated using an enzymatic colorimetric assay based on the cholesterol oxidase method after removal of the other lipoproteins by precipitation with phosphotungstate‐magnesium, following the manufacturer's instructions. The absorbance was measured at 600 nm using an Ultrospec *2000* UV/VIS spectrophotometer (Biochrom Ltd, Cambridge, UK).

#### Determination of serum VLDL‐ and LDL‐cholesterol

Serum VLDL‐ and LDL‐cholesterol were calculated indirectly by the Friedewald's equations (Friedewald et al. 1972).

VLDL=Triglycerides/5

LDL=Total‐cholesterol‐ [HDL + VLDL]

#### Determination of atherogenic and coronary risk indices

Atherogenic index (*AI*) and coronary risk index (*CRI*) as measures of the extent of atherosclerotic lesions and coronary atherosclerosis development, respectively, were calculated using serum lipids of different groups of rats using the mathematical formulae (Subramaniam et al. 2011)

AI=[Total cholesterol ‐ HDL]/HDL

CRI=[Total cholesterol]/HDL

### Data and statistical analysis

All the results are expressed as means ± SEM. To examine the quantitative differences among the experimental groups, the respective data were subjected to analysis of variance (ANOVA) using the GraphPad Prism‐ 5.03 (GraphPad Software Inc., California, CA) statistical programme. *Post hoc* comparisons were made using Tukey's multiple comparisons. In all tests, *P *< 0.05 value was used as the criterion for statistical significance.

## Results

### Body weight data

**Figure 2:**
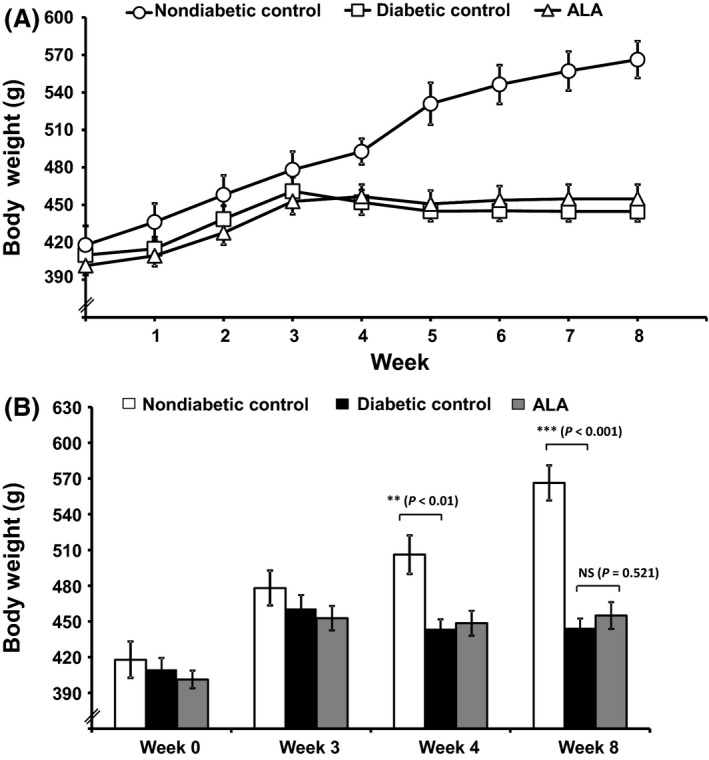
Effect of ALA on HFD and STZ‐induced body weight change in rats. Daily recordings of the mean body weight changes of the experimental groups of rats (A) and comparison of the mean body weights of rats at weeks 0, 3, 4, and 8 among the different groups of rats (B). Treatment with ALA for 4 weeks (from week 4 to week 8) protected the diabetes‐induced weight loss (nonsignificant) as compared to diabetic control rats. Each bar represents the mean ± SEM of 5–10 rats. **P < 0.01 and ***P < 0.001 when compared with diabetic control group. NS, No significant difference between the groups in comparison.

The changes in the mean body weight of the different groups of rats over the 8‐weeks study period are shown in Figure 2A. There was no significant difference in the initial body weights (from 401.3 ± 7.6 g to 417.9 ± 15.2 g; *n* = 5–10) and body weights after 3 weeks between different groups. However, 1 week post‐STZ injection (week 4), diabetic control rats displayed a significant (*P *< 0.01) reduction in body weight by 8.13% compared with the non‐diabetic control rats. After 8 weeks, however, this difference was more pronounced by 21.55% and gained higher significance (*P *< 0.001). On the other hand, *ALA*‐treated rats showed a tendency to reverse the STZ‐induced body weight reduction, although this change was not significant (454.9 ± 11.3 g vs. 444.7 ± 7.9 g; *P* = 0.687) when compared with the diabetic control after 8 weeks (Fig. 2B).

### Serum glucose

As shown in Table 1, diabetic control rats that received HFD and low‐dose STZ showed a significant (*P *< 0.001; *n* = 10) 3.1‐fold elevation of fasting blood glucose after 8 weeks compared with the non‐diabetic control rats (*n* = 5). However, *ALA* treatment (100 mg/kg body weight) for 4 weeks produced a significant (*P *< 0.001; *n* = 9) 2.4‐fold decrease in blood glucose compared with the diabetic control rats (Table 1).

**Table 1:** Effect of alpha‐lipoic acid on high‐fat diet (HFD) and streptozotocin (STZ)‐induced metabolic derangements of glucose, triglycerides, and insulin sensitivity in rats

Group	Serum parameters		
	Glucose (mg/dL)	Triglycerides (mg/dL)	TyG Index
Non‐diabetic control	115.8 ± 7.88	68.5 ± 5.39	8.2 ± 0.06
Diabetic control	479.9 ± 14.03###	107.8 ± 2.50###	10.1 ± 0.03###
ALA (100 mg/kg)	195.7 ± 11.00***	86.8 ± 2.01***	9.0 ± 0.05***

ALA: alpha‐lipoic acid; HFD: high‐fat diet; STZ: streptozotocin.

Values represent the mean ± SEM of 6–12 rats (*n* = 5–10). Significant difference from diabetic control: ***P < 0.001.

Significant difference from non‐diabetic control: ^###^ *P* < 0.001.

### Serum triglycerides

The levels of serum triglycerides in the different groups of rats are shown in Table 1. The diabetic control rats exhibited a significant (*P *< 0.001; *n* = 10) increase in serum triglycerides levels after 8 weeks compared with the non‐diabetic control rats (*n* = 5). However, the rats treated with *ALA* (100 mg/kg) for 4 weeks significantly (*P *< 0.001; *n* = 9)) reduced the serum triglyceride level (86.8 ± 2.0 mg/dL vs. 107.9 ± 2.5 mg/dL) by 1.2‐fold compared with the diabetic control rats (*n* = 10).

### TyG‐index

The effect of *ALA* on insulin sensitivity as assessed from the *TyG*‐index in the different groups of rats is shown in Table 1. Diabetic control rats that received HFD and low‐dose STZ showed a significant (*P *< 0.001; *n* = 10) reduction of insulin sensitivity when compared with the non‐diabetic control rats (*n* = 5) after 8 weeks. On the other hand, the rats treated with *ALA* (100 mg/kg) showed a significant (*P *< 0.001; *n* = 9) increase in insulin sensitivity (9.0 ± 0.05 vs. 10.1 ± 0.03) as measured from *TyG‐*index compared with the diabetic control rats (*n* = 10).

### Serum total‐cholesterol

**Figure 3:**
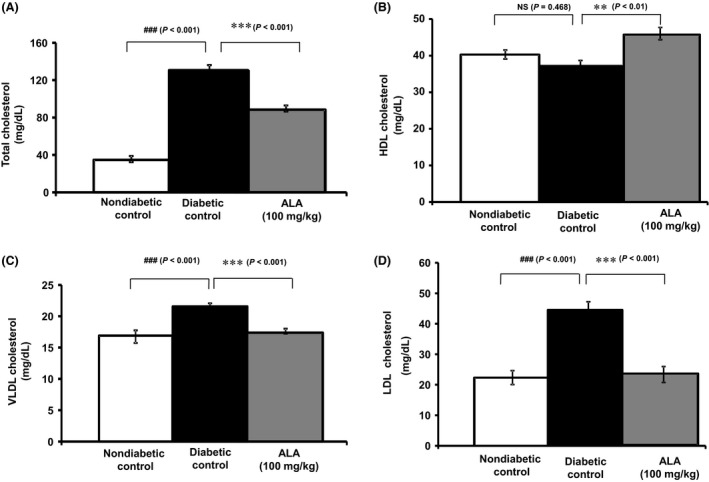
Effect of ALA on high‐fat diet and STZ‐induced metabolic derangements of lipid profiles. Each bar represents the mean ± SEM of 5–10 rats. **P < 0.01 and ***P < 0.001 when compared with diabetic control group. ###*P *< 0.001 when compared with non‐diabetic control group. NS, No significant difference between the groups in comparison.

Figure 3A shows the total cholesterol levels among the various experimental groups. Diabetic rats (*n* = 10) that received HFD and low‐dose STZ showed a significant (*P *< 0.001) 2.8‐fold elevation of total‐cholesterol levels after 8 weeks compared with the non‐diabetic control rats (*n* = 5). However, *ALA* treatment (100 mg/kg) for 4 weeks produced a significant (*P *< 0.001; *n* = 9) 1.5‐fold decrease in serum total‐cholesterol (88.4 ± 3.1 mg/dL vs. 130.6 ± 3.8 mg/dL) compared with the diabetic control rats (*n* = 10).

### Serum HDL‐cholesterol

As depicted in Figure 3B, diabetic rats (*n* = 10) that received HFD and low‐dose STZ tended to show a decrease in the levels of HDL‐cholesterol although not significant (*P* = 0.468) when compared with the non‐diabetic control rats (*n* = 5). Interestingly, *ALA* treatment (100 mg/kg) for four weeks showed a significant (*P *< 0.01, *n* = 9) 1.2‐fold increase in HDL‐cholesterol levels (45.8 ± 1.7 mg/dL vs. 37.2 ± 1.7 mg/dL) compared with the non‐diabetic control rats (*n* = 10).

### Serum VLDL‐ and LDL‐cholesterol

The levels of VLDL‐ and LDL‐cholesterol as calculated by Friedewald's equation in the various groups of experimental rats are shown in Figure 3C and D respectively. Diabetic control rats (*n* = 10) showed a significant elevation of serum VLDL‐cholesterol (0.2 fold; *P *< 0.001) and LDL‐cholesterol (0.9 fold; *P *< 0.001) after 8 weeks compared with the non‐diabetic control rats (*n* = 5). A 0.19‐fold decrease in VLDL‐cholesterol levels was observed in rats treated with *ALA* (100 mg/kg) which reached statistical significance (*P *< 0.001, *n* = 9) when compared with the diabetic control rats after 4 weeks of treatment. In parallel, a 0.9‐fold decrease in LDL‐cholesterol levels was observed in rats treated with *ALA* (100 mg/kg) which reached statistical significance (*P *< 0.001, *n* = 9) when compared with the diabetic control rats after 4 weeks of treatment.

### Atherogenic index and coronary risk index

**Figure 4:**
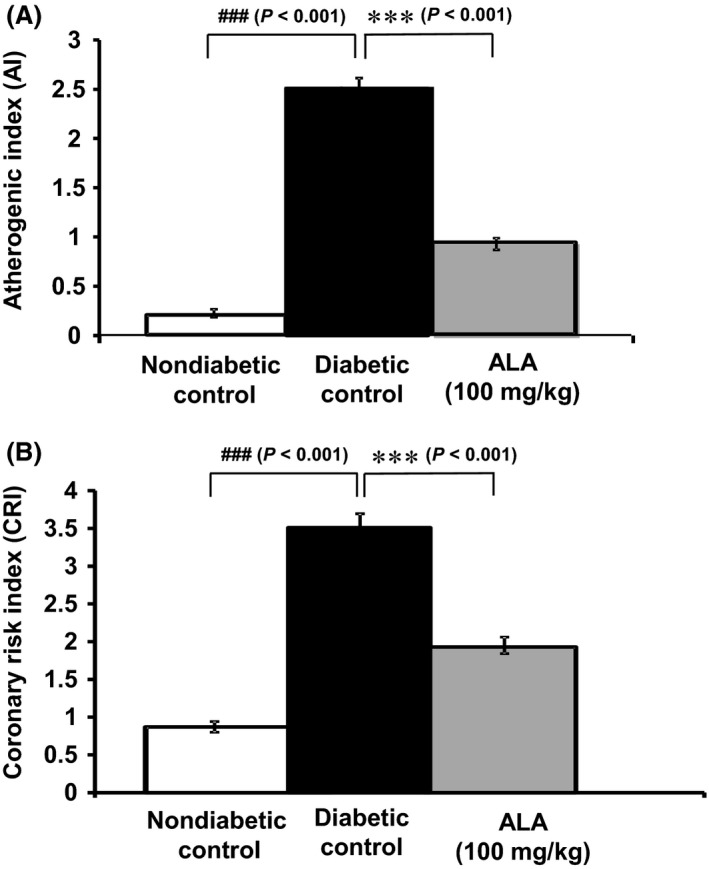
Effect of ALA on high‐fat diet and STZ‐induced atherogenic (A) and coronary risk indices (B). All bars represent Mean ± SEM of 5–10 rats (*n* = 5–10). ***P < 0.001 when compared with diabetic control group. ###*P *< 0.001 when compared with non‐diabetic control group. NS, No significant difference between the groups in comparison.

The effect of *ALA* on reducing the risk of developing atherosclerotic lesions and coronary atherogenesis as assessed from *AI* and *CRI* in the different groups of rats is shown in Figure 4. Diabetic control rats that received HFD and low‐dose STZ showed a significant (*P *< 0.001, *n* = 10) elevation of *AI* and *CRI* after 8 weeks compared with non‐diabetic control rats (*n* = 5). The rats treated with *ALA* (100 mg/kg) displayed a significant (*P *< 0.001) reduction in the risk of developing atherosclerotic lesions (Fig. 4A) and coronary atherogenesis (Fig. 34B) as measured from the *AI* (2.51 ± 0.21 vs. 0.93 ± 0.61) and *CRI* (3.51 ± 0.21 vs. 1.93 ± 0.11) when compared with the non‐diabetic control rats.

## Discussion

Metabolic syndrome, a concurrence of disorders including obesity, impaired fasting glucose and/or impaired glucose tolerance, reduced insulin sensitivity, hyperinsulinemia, dyslipidemia, and hypertension has gained importance because of its association with the subsequent development of *T2D* and its complications (Fujimoto 2000). The development of metabolic syndrome is influenced by a combination of genetic and environmental factors. Among the environmental factors, long‐term high‐fat intake (which reflects the current day living style) is most intensively studied because of its contribution to the development of metabolic syndrome in humans and rodents (Buettner et al. 2006). When carbohydrates are in low supply, or their breakdown is incomplete, fats become the preferred source of energy (Edelman 1998). As a result, fatty acids are mobilized into the general circulation, leading to secondary triglyceridemia whereby the total serum lipids, including triglycerides and cholesterol are increased. Consequently, this will result in abnormal blood lipid profiles that lead to life‐threatening disorders (Buettner et al. 2007; Fernandez 2007).

In this study, we examined the curative effects of *ALA* in HFD‐fed plus low‐dose STZ injected rats, a metabolic model of dyslipidemia, insulin resistance and *T2D* that is similar to human metabolic syndrome (Skovsø 2014). Since rats have similar metabolic patterns as that of human beings, it is rational to use this disease model to examine the curative effects of chronic treatment of *ALA*. Although there are a variety of animal models (spontaneous as well as disease‐induced) available for the study of metabolic syndrome and *T2D*, the pattern of disease initiation and development in most of the models do not appear to be closely analogous to the clinical situation in humans (Srinivasan and Ramarao 2007). Models such as the Zucker diabetic fatty rat, ob/ob mouse, and db/db mouse develop diabetes genetically, unlike in humans (Fröde and Medeiros 2008). In HFD‐fed animal models, the development of obesity, dyslipidemia, hyperinsulinemia, and insulin resistance are observed but not hyperglycemia or *T2D*, thus limiting the screening of agents that control blood glucose in *T2D* (Reuter 2007). In contrast, the chemical‐induced (STZ or alloxan) animal models require relatively high doses of STZ or alloxan and the development of hyperglycemia in these models is primarily due to direct pancreatic beta cell destruction, resulting in insulin deficiency rather than as a consequence of insulin resistance (Lenzen 2008). Thus, the chemical‐induced models represent the symptoms and characteristics typical of human *T1D* than *T2D* and consequently, are not very responsive to insulinotropic and insulin‐sensitizing compounds. On the other hand, the HFD‐fed and low‐dose STZ‐induced rat model initially developed by Reed et al. (2000) and modified by Srinivasan et al. (2005) closely mimics the natural history of human metabolic syndrome and *T2D*. This model offers many advantages over the afore‐mentioned stand‐alone disease induction with either HFD or STZ in that short‐term feeding (3 weeks) with HFD develops dyslipiemia and insulin resistance as seen in metabolic syndrome, and injection of low‐dose STZ (35 mg/kg, i.p.) induces partial destruction of beta cells to suppress insulin secretion with a net result of persistent hyperglycaemia as seen in *T2D*. To the best of our knowledge, this is the first study to use HFD‐fed and low‐dose STZ‐induced rat model in investigating the curative effects of *ALA* on in vivo glucose and lipid homeostasis.

In this study, the *TyG* index was evaluated as a surrogate method for estimation of insulin sensitivity (Simental‐Mendia et al. 2008). The combination of HFD‐feeding and low‐dose STZ injection significantly decreased the body weight and insulin sensitivity and increased serum glucose in rats. The study revealed that *ALA* (100 mg/kg) effectively lowered blood glucose and increased insulin sensitivity in the rat model, and this was found to be consistent with previous in vivo reports (Khamaisi et al. 1999; Budin et al. 2007; Chen et al. 2009; Salama 2011; Sudheesh et al. 2011; Arambasic et al. 2013; Dinic et al. 2013). Nagamatsu et al. (1995) reported that normal rats chronically treated with 100 mg/kg *ALA* showed a marked reduction in blood glucose which also supports an earlier observation by Obrosova et al. (1998) in STZ‐induced diabetic rats. Clearly, the results of these earlier observations substantiate our findings in the HFD‐fed and low‐dose STZ‐induced rats.

It has been demonstrated that the molecular actions of ALA either directly or indirectly (through cellular redox status) on the insulin signaling pathway are responsible for its glucose lowering and insulin‐sensitizing properties (Gomes and Negrato 2014; Rochette et al. 2015). Earlier studies have shown that *ALA* increases glucose uptake in insulin‐sensitive (Khanna et al. 1999) and insulin‐resistant tissues (Streeper et al. 1997). Later studies by Yaworsky et al. (2000) and Konrad et al. (2001) observed that *ALA* stimulates tyrosine phosphorylation of insulin receptor substrate (IRS‐1) and stimulates expression, translocation and intrinsic activity of glucose transporters (GLUTs) leading to increased cellular glucose uptake Further studies have revealed that *ALA* ameliorates impaired glucose metabolism and insulin resistance and increases energy expenditure in peripheral tissues by activating 5′‐adenosine monophosphate‐activated protein kinase (AMPK) directly (Lee et al. 2005) or indirectly (Shen et al. 2007) through Ca/calmodulin‐dependent protein kinase (CaMKK). This leads to increased activity of peroxisome proliferator‐activated receptor‐gamma coactivator‐1alpha (PGC‐1 *α*) (Wang et al. 2010). Thus, the constellation of these findings strongly support the results of our present study by suggesting that glucose‐regulating and insulin‐sensitizing effects of *ALA* could be at least, in part, be attributed to a direct stimulatory effect on glucose transporters and/or indirect stimulation of tyrosine and serine/threonine kinases phosphorylation and/or increased AMPK activation.

Dyslipidemia is the most important modifiable risk factor contributing to the development of atherosclerosis in *T2D* (Niemeijer‐Kanters et al. 2001). Thus the importance of blood levels of triglycerides and cholesterol in the pathogenesis of lipid disorders has been extensively reviewed (Raal 2009). In this study, the combination of HFD‐feeding and low‐dose STZ injection markedly increased the serum triglycerides, total‐, VLDL‐ and LDL‐cholesterol with a tendency to decrease HDL‐cholesterol. Presumably, these changes may have occurred, in part, by enhanced cholesterol biosynthesis resulting from the high fat diet (Jones 1997). The high levels of LDL‐cholesterol may have been due to reduced expression or activity of the LDL‐receptor sites in response to the high‐fat diet treatment (Brown and Goldstein 1986). Therefore, interventions aimed at lowering the triglycerides and LDL‐cholesterol and/or raising the HDL‐cholesterol may be an important strategy in lowering the serum triglycerides and total cholesterol in rats fed a high‐fat diet. Consistent with earlier reports, *ALA* administration along with the high‐fat diet significantly reduced the serum total cholesterol, LDL‐cholesterol, VLDL‐cholesterol and serum triglycerides in addition to marked improvement of HDL‐cholesterol in vivo (Butler et al. 2009; Seo et al. 2012).

An earlier study demonstrated that *ALA* reduces the activity of HMG‐CoA reductase (involved in cholesterol biosynthesis), and increases the activities of lipoprotein lipase (involved in triglyceride turnover) and lecithin cholesterol acyl transferase (LCAT; involved in HDL maturation) (Thirunavukkarasu et al. 2004). Furthermore, *ALA* has been shown to increase the expression of apolipoprotein‐A (involved in reverse cholesterol transport) LDL receptor (involved in liver uptake of LDL‐cholesterol) in the liver (Marangon et al. 1999). These observations were substantiated by a recent study by Carrier et al. (2014) demonstrating that the increased expression of the LDL receptor in the liver upon chronic *ALA* supplementation in HFD‐fed Zucker obese rats is associated with a reduced gene expression and serum levels of proprotein convertase subtilisin/kexin type 9 (PCSK9; involved in LDL receptor degradation). In addition, recent observations from in vitro and in vivo studies have demonstrated that *ALA* activates both SIRT1 and AMPK which regulate acetyl‐CoA carboxylase (ACC), adipose triacylglycerol lipase (ATGL) and fatty acid synthase (FAS) (Chen et al. 2012). Moreover, it has also been demonstrated that *ALA* decreases hepatic lipogenesis by suppressing the expression of lipogenic genes carbohydrate‐responsive element‐binding protein (ChREBP) and sterol‐responsive element‐binding protein‐1c (SREBP‐1c) in the liver (Castro et al. 2013). Yang et al. (2008) also reported that lipid reduction in response to *ALA* treatment is associated with an increase in the expression of lipolytic genes carnitine palmitoyltransferase‐1 (CPT‐1), peroxisome proliferator‐activated receptor‐alpha (PPAR‐ *α*), and acyl‐CoA oxidase (ACOX). Thus, the molecular mechanisms responsible for the observed lipid‐lowering effects of *ALA* could be due to multiple effects on potential sites of action, leading to decreased intestinal fat absorption and/or decreased lipid biosynthesis and/or enhanced lipid metabolism.

Long‐term dyslipidemia associated with *T2D* increases the risk of development of atherosclerosis (Susanti et al. 2010). In particular, the oxidative modification of LDL cholesterol is implicated in the formation of atherosclerotic plaque in the blood vessels. The atherogenic index (*AI*), which indicates the deposition of foam cells or fatty plaque deposition in blood vessels, is positively correlated to the development of cardiovascular disease (Kwiterovich 2000). Thus, *AI* is an important predictor of coronary risk potential as it gives an indirect measure of LDL and HDL lipoprotein particle size. Consistent with earlier reports, *ALA* treatment significant reduced *AI* in the HFD‐fed and STZ‐induced T2D rats which corresponds to decreased LDL‐cholesterol and increased HDL‐cholesterol (Amom et al. 2008; Seo et al. 2012). There is strong evidence suggesting a direct correlation between high circulating total cholesterol levels and low HDL‐cholesterol levels, with the increased risk of coronary artery disease (Stratton et al. 2000). Upon *ALA* treatment, the total‐cholesterol: HDL‐cholesterol ratio, as a measure of Framingham's coronary risk index (*CRI*), is markedly reduced in the present study. This directly corresponds to decreased total‐cholesterol and increased HDL‐cholesterol (D'Agostino et al. 2008). Thus, the significant reduction of the *AI* and *CRI* scores clearly demonstrate the potential of *ALA* supplementation on reducing the risk of cardiovascular complications of *T2D*.

In summary, the present investigation suggests that *ALA* has a protective effect on the high‐fat diet and low‐dose STZ‐induced metabolic disturbances by strongly suppressing the hyperglycemic and hyperlipidemic conditions. Thus, the present findings emphasize that *ALA* supplementation is beneficial in the prevention of metabolic disorders caused by a high‐fat diet. Further clinical studies are warranted to explain the mechanism(s) of the glucose and lipid metabolism‐regulating activities of *ALA*.

## Disclosure

The authors declare that there are no known conflicts of interest associated with this publication and there has been no direct or indirect financial support or no non‐financial support for the subject matter or materials discussed in this work that could influence its outcome.
